# High Burden of Antimicrobial Resistance among Bacteria Causing Pyogenic Wound Infections at a Tertiary Care Hospital in Kathmandu, Nepal

**DOI:** 10.1155/2017/9458218

**Published:** 2017-08-28

**Authors:** Basista Prasad Rijal, Deepa Satyal, Narayan Prasad Parajuli

**Affiliations:** Division of Clinical Microbiology, Department of Clinical Laboratory Services, Manmohan Memorial Medical College and Teaching Hospital, Kathmandu, Nepal

## Abstract

Pyogenic wound infections are one of the most common clinical entities caused and aggravated by the invasion of pathogenic organisms. Prompt and aggressive antimicrobial therapy is needed to reduce the burden and complications associated with these infections. In this study, we intended to investigate the common pathogens and their antimicrobial susceptibility patterns from the pyogenic wound infections at a tertiary care hospital in Kathmandu, Nepal. A laboratory based cross-sectional study was carried out among the pyogenic clinical specimens of the patients visiting Manmohan Memorial Teaching Hospital, Kathmandu, Nepal. Processing of clinical specimens and isolation and identification of bacterial pathogens were carried out using standard microbiological methods. Antimicrobial susceptibilities and resistant profiles were determined by following the standard guidelines of Clinical and Laboratory Standards Institute (CLSI). About 65% of the clinical specimens were positive for the bacterial growth and Gram positive bacteria (57.4%) were the leading pathogens among pyogenic wound infections.* Staphylococcus aureus* (412, 49.28%),* Escherichia coli* (136, 16.27%),* Klebsiella* spp. (88, 10.53%), and* Pseudomonas* spp. (44, 5.26%) were the common pathogens isolated. High level of drug resistance was observed among both Gram positive bacteria (51.9%) and Gram negative bacteria (48.7%). Gram positive isolates were resistant to ampicillin, ciprofloxacin, cotrimoxazole, erythromycin, and cloxacillin. Gram negative isolates were resistant to cephalosporins but were well susceptible to amikacin and imipenem. Pyogenic wound infections are common in our hospital and majority of them were associated with multidrug resistant bacteria. The detailed workup of the prevalent pathogens present in infected wounds and their resistance pattern is clearly pertinent to choosing the adequate treatment.

## 1. Background

Infections of the skin and soft tissue due to either trauma, surgery, or burns may result in the generation of exudates composed of dead leucocytes, cellular debris, and necrotic tissues [1]. Pyogenic or pus forming wound infections are characterized by severe local inflammation subsequent to tissue injury leading to generalized clinical disease through the various toxic mechanisms associated with invasion of pyogenic bacteria. Some of the common etiological agents responsible for causing pyogenic infections are bacteria such as* Staphylococcus aureus*,* Streptococcus pyogenes*,* Escherichia coli*,* Klebsiella *spp.,* Proteus *spp.,* Pseudomonas *spp., and* Acinetobacter *spp. [2, 3]. The profusion and diversity of organisms are principally influenced by predisposing conditions, anatomic location of infection including its type, quality, and level of tissue perfusion, and antimicrobial efficacy of the host response [4].

Pyogenic wound infections are significant subgroup of infections encountered by infectious disease physicians in the hospitals worldwide. These infections are associated with higher morbidity and therefore antimicrobial regimens are generally recommended to reduce the burden as well as to prevent associated long term complications [5]. Moreover, surgical drainage is also required in severe closed type wound infections [6]. Despite the advancements in diagnostic techniques, treatment of pyogenic infections in the developing countries is challenging due to the emergence of multidrug resistant (MDR) pathogens. In particular, a large number of methicillin resistant* Staphylococcus aureus* along with the multidrug resistant Gram negative isolates are increasingly allied with pyogenic infections in recent years [7]. The crisis of antibiotic resistance among pyogenic bacterial infections has been attributed to the inappropriate use of antimicrobial agents particularly in developing country [8].

The antimicrobial resistance has become a global challenge and the resistant pathogen poses a grave threat to the public health worldwide. Different studies are being conducted across the globe to access the bacterial profile in pyogenic wound infection [3]. However, in Nepal, the studies are not consistent enough to reveal the information regarding pyogenic pathogens including their antibiotic susceptibility pattern [9, 10]. The appropriate knowledge of the pathogens, their resistant character, and their updated antimicrobial therapy plays a crucial role in the treatment process as well as in infection control measures. Therefore, this study was intended to characterize the bacterial isolates from clinical specimens of pyogenic wound infections and to determine the antibiotic susceptibilities to commonly used therapeutic regimens at a tertiary care hospital of Kathmandu.

## 2. Methods

### 2.1. Study Design and Samples

A laboratory based cross-sectional study was carried out at the Department of Clinical Microbiology of Manmohan Memorial Teaching Hospital, Kathmandu, Nepal, from April 2016 to March 2017 (over the period of one year). Clinical specimens such as pus, wound aspirate, wound swab, necrotic tissue, and surgical drainage were collected aseptically from suspected patients with pyogenic wound infections and processed in the microbiology laboratory with minimal delay. However, the specimens not fulfilling the criteria of American Society for Microbiology (ASM) [11] and duplicate specimens from same patients were excluded from this study. During the study period, a total of 1,198 specimens representing the pyogenic infections were processed.

### 2.2. Laboratory Methods

Each aseptically collected specimen was inoculated onto the Blood Agar (BA), Chocolate Agar (CA), and MacConkey Agar (MAC) plates (HiMedia Laboratories, India) by surface streaking method. BA and MAC plates were incubated in aerobic atmosphere and CA plates were incubated in additional 5–10% CO_2_ at 37°C for 24–48 hours. Identification of significant isolates associated with pyogenic infections was carried out following standard microbiological techniques including morphological appearance of the colonies: Gram's staining, catalase test, coagulase test, and oxidase test with other biochemical parameters [11]. Assurance of pure culture inoculum was done by setting purity plate along with the biochemical tests.

### 2.3. Antimicrobial Susceptibility Testing

The susceptibility of bacterial isolates against different antibiotics was determined by the disk diffusion method [modified Kirby-Bauer method] on Mueller Hinton agar (HiMedia, India) following standard procedures recommended by the Clinical and Laboratory Standards Institute (CLSI), Wayne, USA [12]. For this purpose, the following antibiotics with specified concentrations were used: ampicillin (10 *μ*g), trimethoprim-sulfamethoxazole/cotrimoxazole (25 *μ*g), gentamycin (10 *μ*g), high level gentamycin (120 *μ*g), amikacin (30 *μ*g), ciprofloxacin (5 *μ*g), levofloxacin (5 *μ*g), cefoxitin (30 *μ*g), cefotaxime (30 *μ*g), ceftazidime (30 *μ*g), cloxacillin (5 *μ*g), erythromycin (15 *μ*g), clindamycin (2 *μ*g), imipenem (10 *μ*g), vancomycin (30 *μ*g), teicoplanin (30 *μ*g), piperacillin-tazobactam (100/10 *μ*g), polymixin B (300 units), and colistin sulphate (10 *μ*g) from HiMedia Laboratories, India. Interpretations of antibiotic susceptibility results were made according to the guidelines of interpretative zone diameters of CLSI [12].* Escherichia coli* ATCC 25922,* Staphylococcus aureus* ATCC 25923, and* Pseudomonas aeruginosa* ATCC 27853 were used as the control organisms for antibiotic sensitivity.

### 2.4. Identification of Multidrug Resistant (MDR) Isolates

Multidrug resistant (MDR) bacterial isolates were identified according to the criteria recommended by international expert committee of the European Centre for Disease Prevention and Control (ECDC) and the Centers for Disease Control and Prevention (CDC) [13]. In this study, the isolate resistant to at least one antimicrobial from three different groups of first-line drugs tested was regarded as multidrug resistant (MDR).

### 2.5. Phenotypic Test for Methicillin Resistant (MRSA) and Inducible Clindamycin Resistant (iMLS_B_)* Staphylococcus aureus*

Methicillin resistant* Staphylococcus aureus* (MRSA) isolates were detected by cefoxitin disk (30 *μ*g) method of CLSI.* S. aureus* isolates were judged as methicillin resistant when the ZOI for cefoxitin was ≤21 mm [12]. Similarly, inducible macrolide-lincosamide streptogramin-B (iMLS_B_) resistance was detected in* S. aureus* by disk approximation using clindamycin (2 *μ*g) and erythromycin (15 *μ*g) on MHA plates. After overnight incubation, isolates with flattened zone of inhibition adjacent to the erythromycin disk (referred to as a “D” zone) were considered to exhibit inducible clindamycin resistance [12].

### 2.6. Ethical Consideration

Written approval (Ref Number 12/MMIHS/2072) was obtained from Institutional Review Committee of Manmohan Memorial Institute of Health Sciences (IRC-MMIHS) after submitting and presenting the research proposal. In addition, informed oral consent was taken from every patient for participation in this study.

### 2.7. Data Processing and Analysis

Data regarding patient demographics, bacterial isolates, antimicrobial susceptibilities, and resistance determinants were entered into a computer program. Data were analyzed using SPSS 20.0 version and interpreted according to frequency distribution, percentage.

## 3. Results

During the study period, 1,198 specimens were processed at the clinical microbiology laboratory; 778 (64.9%) of them showed the significant bacterial growth confirming the infection. In this study, males (66.3%, 515/794) and the patients with age group 15–44 years (44.7%, 348/778) were more affected with pyogenic wound infections (data not presented). Among growth positive specimens, monomicrobial and polymicrobial growth were observed in 720 (92.54%) and 58 (7.46%) specimens, respectively. A total of 836 bacterial pathogens were recovered with the predominance of Gram positive bacteria (480, 57.6%).* Staphylococcus aureus* (412, 49.28%) was the leading bacterial pathogen followed by coliforms* Escherichia coli* (136, 16.27%) and* Klebsiella* spp (88, 10.53%) and Gram negative nonfermenters* Pseudomonas aeruginosa* (44, 5.26%) and* Acinetobacter* spp. (20, 2.39%) (Table 1).

### 3.1. Antibiotic Susceptibilities of Gram Positive Bacteria

Among 14 various antimicrobial agents tested, the susceptibility pattern of Gram positive bacterial isolates varied according to the species. Majority of the isolates (51.9%) were multiple drug resistant (resistant to two or more classes of antimicrobials). Isolates of* Staphylococcus aureus* were highly resistant to ampicillin (83%) and ciprofloxacin (56%) while being less resistant to cotrimoxazole (46%), erythromycin (46%), cloxacillin (37%), clindamycin (32%), and imipenem (32%). Similarly, isolates of* Enterococcus *spp. were resistant to ampicillin (56%) but were highly susceptible to piperacillin-tazobactam, imipenem, and gentamycin (89% each). On the other hand, isolates of* Streptococcus pyogenes* were highly susceptible to ampicillin (88%), ciprofloxacin (90%), erythromycin (96%), clindamycin (96%), and cotrimoxazole (82%). Vancomycin, teicoplanin, and amikacin were the most effective antimicrobials for Gram positive bacteria in our study (Table 2).

### 3.2. Antibiotic Susceptibilities of Gram Negative Bacteria

Diverse susceptibility pattern was observed among the isolates of Gram negative bacteria (Table 3). Enterobacteriaceae isolates were highly resistant to ampicillin (93%) and cephalosporins (up to 68%). Gentamycin, levofloxacin, piperacillin-tazobactam, and imipenem were the effective antimicrobials for enterobacterial strains. On the other hand, Gram negative nonfermenters,* Pseudomonas* and* Acinetobacter* spp., were highly resistant to cephalosporins (up to 66%) but well susceptible to levofloxacin, piperacillin-tazobactam, and imipenem. Polymixin and colistin sulphate were completely effective against Gram negative bacteria.

### 3.3. Multidrug Resistant Gram Negatives and Methicillin Resistant* Staphylococcus aureus*

In this study, we observed high rates of MDR bacteria associated with pyogenic infections. Among Gram negatives, highest MDR strains were* Escherichia coli* (66.18%) followed by* Acinetobacter* spp. (60%),* Klebsiella* spp. (50.0%),* Pseudomonas* spp. (45.45%),* Proteus* spp. (36.84%), and* Citrobacter* spp. (33.33%). Among Gram positives, 49.5% of* Staphylococcus aureus* were MDR and 31.56% isolates were methicillin resistant (MRSA). Furthermore, 8.73% of* S. aureus* isolates were inducible clindamycin resistant (iMLS_B_).

## 4. Discussion

Every year, millions of people in developing countries like Nepal are experiencing the pyogenic wound infections due to either injury related to trauma, accidents, or burns and their complications with pathogenic microorganisms [2, 5, 9]. Although pyogenic wound infections are common findings among the patients visiting hospitals of Nepal, there is paucity of documented reports describing the etiological spectrum and antibiotic susceptibility pattern of bacteria causing these infections [9, 10]. Moreover, continuous upsurge of antimicrobial resistance among the pathogenic organisms has created a therapeutic challenge for treatment of pyogenic wound infections [14]. Therefore, updated knowledge on the etiology and antimicrobiogram is considered highly valuable to reduce morbidities and associated complications.

In this study, overall pyogenic wound infections among study subjects based on the significant bacterial growth in clinical specimens were 64.9%. To the best of our knowledge, this is the highest ever reported rate of growth among the pyogenic clinical specimens from Nepal. Previously, Shrestha and Basnet (50.0%) and Acharya et al. (50.7%) from nearby hospitals have documented quite lower rates of growth among pyogenic clinical specimens [2, 15]. However, our rate of growth is comparable to the previous reports of Rai et al. (59%) [9], Trojan et al. (60.1%) from India [7], and Bessa et al. (69.5%) from Italy [16]. Alongside, extremely high rates of growth among pyogenic clinical specimens were reported by Mohammed et al. (83.9%) and Mama et al. (87.4%) from Ethiopia [3, 17]. These variations in the growth rates from pyogenic wound specimens might be attributable to the quality of specimens processed, contamination with external microbiota, and standard wound care practices in the healthcare and facilities of bacterial cultivation in the locality [16]. Moreover, we noticed that majority (92.54%) of the clinical specimens were found with monomicrobial growth and little polymicrobial growth. This is also consistent with the previous reports from Nepal [2, 15]. Polymicrobial pyogenic wound infections might be associated with poor wound care, increased microbial survival, and ineffective antimicrobial treatment [17].

Gram positive bacteria have been described as the major cause for pyogenic wound infections in several literatures [2, 9, 10]. Our findings also supported this fact, as majority of our isolates were Gram positive cocci (57.6%). However, Trojan et al. from India, Mama et al. from Ethiopia, and Bessa et al. from Italy have documented the Gram negative bacterial dominance in pyogenic wound infections [7, 16, 17]. On the other hand,* Staphylococcus aureus* (49.2%) was the predominant isolate responsible for pyogenic wound infections in this study which is quite similar to several previous studies [2, 5, 9]. In a recent report from India, Gram negative bacteria, particularly Enterobacteriaceae, were found as major pathogens [7].* Escherichia coli*,* Klebsiella* spp., and* Pseudomonas* spp. were other common pathogens in our study. It is well known that* S. aureus* and Gram negative bacterial pathogens produce very potent virulence factors, responsible for maintaining the infection and delaying the process of wound healing [16]. Therefore, our results confirm the usual most prevalent microorganisms found in pyogenic wound infections. Nevertheless, Gram negative bacteria have been described to be associated with nosocomial infections and intra-abdominal surgical procedures [18].

High rates of antimicrobial resistance among the pathogenic bacteria associated with the pyogenic infections are major concerns of this study. The prevalence and pattern of antimicrobial resistance among pyogenic bacterial isolates usually exhibit variability according to the geographic areas, climatic conditions, and endemicity of resistant pathogens in the locality. Of particular concern, among Gram positive bacteria,* Staphylococcus aureus* in this study was the most resilient organism to develop resistance. Our isolates were highly resistant to ampicillin, ciprofloxacin, cotrimoxazole, erythromycin, cloxacillin, clindamycin, and imipenem. This finding is in agreement with the previous reports of Acharya et al., Rai et al., and Yakha et al. [2, 9, 10] but higher when compared to the reports by Shrestha and Basnet [15] from nearby hospitals of Kathmandu. However, similar to other previous studies [2, 15], the isolates of* Streptococcus pyogenes* were promisingly susceptible to ampicillin, cotrimoxazole, erythromycin, and cephalosporins. Cotrimoxazole, one of the most widely used antimicrobial agents for treating pyogenic and soft tissue infections, was found susceptible to* S. aureus* and* S. pyogenes* [19]. However, isolates of* Enterococcus* spp. were least susceptible to ampicillin, the drug of choice for enterococcal infections [20]. Remarkable susceptibility of Gram positive bacteria to vancomycin, amikacin, and carbapenems (imipenem) may be the good alternative for pyogenic wound infections in our settings.

Furthermore, almost more than half (52%) of the Gram positive strains in our study were MDR which is comparatively higher than that of previous reports from Nepal [2, 10]. Higher rates of MDR strains have been documented in several other studies [17, 21–23]. We believe our rate of MDR Gram positive isolates is greatly contributed by the high rates of methicillin resistant* Staphylococcus aureus* (MRSA) strains [24]. In this study, about 32% of the* Staphylococcus aureus* isolates were methicillin resistant and were resistant to commonly used antimicrobial agents. The MRSA rate is high when compared to the previous reports of Acharya et al. (22.5%) [2] and Rai et al. (19%) [9] but is lower when compared to the reports of Belbase et al. (47.4%) and Khanal et al. (68%) [14, 25]. In addition to this, similar to previous studies [14, 26], we found inducible clindamycin resistance (iMLS_B_) in 8.73% of the isolates. The possible explanation for variation in the drug susceptibilities might be difference in study population including hospitalized inpatients where more MDR strains are expected.

Alongside, our findings indicate the high incidence of drug resistance among Gram negative isolates too. In this study,* Escherichia coli*,* Klebsiella* spp.,* Citrobacter* spp., and* Proteus* spp. were highly resistant to cephalosporins while Gram negative nonfermenters were resistant to fluoroquinolones, aminoglycosides, and cephalosporins. The findings of the susceptibility pattern of our Gram negative isolates are in agreement with other previous reports from this region [2, 9, 15]. In recent years, there is an increased concern about Gram negative resistance to commonly used antimicrobials in wound infections [7, 27]. In this study, multidrug resistance among Gram negative bacteria was common where* Escherichia coli* (66.18%),* Acinetobacter* spp. (60%),* Klebsiella* spp. (50.0%), and* Pseudomonas* spp (45.45%) were major MDR strains. This finding is quite high when compared to the previous reports from our country [2, 10] but is lower than that of other studies from India [7] and Ethiopia [22]. The high rates of resistance in Gram negative bacteria in our hospital have been previously found as *β*-lactamase producers [28]. In this scenario, non-*β*-lactam antibiotics including fluoroquinolones and aminoglycosides would be better therapeutic regimens for pyogenic wound infections in our settings.

Our findings indicate the existence of high drug resistant bacteria in pyogenic wound infections. The high use of *β*-lactam antibiotics and inappropriate infection control procedures in the hospitals might be the cause of rising rates of resistance among these bacteria. Moreover, longer duration of prophylactic antimicrobial exposure in surgical interventions may contribute to organisms for developing resistance.

## 5. Limitations

This study was based on characterization of bacterial isolates growing in the aerobic or facultative anaerobic conditions excluding anaerobic bacteria. Furthermore, risk factors for pyogenic wound infections and the treatment outcomes were not measured. Molecular characterization of MDR bacterial isolates would have generated more useful epidemiological results.

## 6. Conclusion

Pyogenic wound infections were mainly caused by* S. aureus*,* Escherichia coli*,* Klebsiella* spp., and* Pseudomonas* spp. High level of drug resistance among both Gram positive and Gram negative bacteria was observed. Continuous surveillance is necessary to update the knowledge of antimicrobial susceptibility profiles of clinical isolates to provide the most appropriate dose regimen and treatment schedule against pyogenic wound infections and to limit the expanding menace of drug resistance.

## Figures and Tables

**Table 1:** Bacterial isolates associated with pyogenic wound infections.

Bacterial isolates	Frequency	%
*Gram positive isolates*	*480*	*57.4*
*Staphylococcus aureus*	412	49.28
*Streptococcus pyogenes*	50	5.98
*Enterococcus* spp.	18	2.15
*Gram negative isolates*	*356*	*42.6*
*Escherichia coli*	136	16.27
*Klebsiella *spp.	88	10.53
*Pseudomonas aeruginosa*	44	5.26
*Proteus *spp.	38	4.55
*Citrobacter *spp.	30	3.59
*Acinetobacter *spp.	20	2.39

*Total*	*836*	*100.0*

**Table 2:** Antibiogram of Gram positive isolates.

Antibiotics	*Staphylococcus aureus* (*n* = 412)		*Streptococcus pyogenes* (*n* = 50)		*Enterococcus* spp. (*n* = 18)	
	Sensitive%	Resistant%	Sensitive%	Resistant%	Sensitive%	Resistant%
Ampicillin	72 (17.48)	340 (82.52)	44 (88.0)	6 (12.0)	8 (44.44)	10 (55.56)
Cotrimoxazole	224 (54.37)	188 (45.63)	41 (82.0)	9 (18.0)	—	—
Gentamycin	348 (84.47)	64 (15.53)	—	—	16 (88.89)	2 (11.11)
Amikacin	383 (92.96)	29 (7.04)	—	—	16 (88.89)	2 (11.11)
Ciprofloxacin	182 (44.17)	230 (55.83)	45 (90.0)	5 (10.0)	14 (77.78)	4 (22.22)
Cefoxitin	282 (68.44)	130 (31.56)	45 (90.0)	5 (10.0)	—	—
Cefotaxime	282 (68.44)	130 (31.56)	45 (90.0)	5 (10.0)	—	—
Cloxacillin	260 (63.11)	152 (36.89)	—	—	—	—
Erythromycin	224 (54.37)	188 (45.63)	48 (96.0)	2 (4.0)	—	—
Clindamycin	279 (67.72)	133 (32.28)	48 (96.0)	2 (4.0)	—	—
Piperacillin + taz.	271 (65.78)	141 (34.22)	—	—	16 (88.89)	2 (11.11)
Imipenem	282 (68.44)	130 (31.56)	—	—	16 (88.89)	2 (11.11)
Vancomycin	412 (100.0)	0 (0.0)	50 (100.0)	0 (0.0)	18 (100.0)	0 (0.0)
Teicoplanin	412 (100.0)	0 (0.0)	50 (100.0)	0 (0.0)	18 (100.0)	0 (0.0)

**Table 3:** Antibiotic susceptibilities of gram negative bacterial isolates.

Antibiotics	*Escherichia coli* (*n* = 136)		*Klebsiella* spp. (*n* = 88)		*Citrobacter *spp. (*n* = 30)		*Proteus *spp. (*n* = 38)		*Pseudomonas *spp. (*n* = 44)		*Acinetobacter* spp. (*n* = 10)	
	*S* (%)	*R* (%)	*S* (%)	*R* (%)	*S* (%)	*R* (%)	*S* (%)	*R* (%)	*S* (%)	*R* (%)	*S* (%)	*R* (%)
Ampicillin	10 (7.3)	126 (92.6)	—	—	—	—	—	—	—	—	—	—
Cotrimoxazole	66 (48.5)	70 (51.4)	44 (50.0)	44 (50.0)	14 (46.6)	16 (53.4)	16 (42.1)	22 (57.9)	—	—	5 (50.0)	5 (50.0)
Gentamycin	94 (69.1)	42 (30.8)	56 (63.6)	32 (36.4)	22 (73.3)	8 (26.7)	22 (57.9)	16 (42.1)	25 (56.8)	19 (43.2)	4 (40.0)	6 (60.0)
Amikacin	118 (86.7)	18 (13.2)	72 (81.8)	16 (18.2)	28 (93.3)	2 (6.7)	29 (76.3)	9 (23.7)	32 (72.7)	12 (27.3)	4 (40.0)	6 (60.0)
Ciprofloxacin	64 (47.0)	72 (53.0)	48 (54.5)	40 (45.5)	16 (53.3)	14 (46.7)	25 (65.8)	13 (34.2)	22 (50.0)	22 (50.0)	5 (50.0)	5 (50.0)
Levofloxacin	100 (73.5)	36 (26.5)	48 (54.5)	40 (45.5)	16 (53.3)	14 (46.7)	25 (65.8)	13 (34.2)	34 (77.2)	10 (22.8)	5 (50.0)	5 (50.0)
Cefotaxime	44 (32.3)	92 (67.7)	42 (47.7)	46 (52.3)	20 (66.7)	10 (33.3)	21 (55.3)	17 (44.7)	15 (34.0)	29 (66.0)	4 (40.0)	6 (60.0)
Ceftazidime	44 (32.3)	92 (67.7)	42 (47.7)	46 (52.3)	20 (66.7)	10 (33.3)	21 (55.3)	17 (44.7)	15 (34.0)	29 (66.0)	4 (40.0)	6 (60.0)
Piperacillin-tazobactam	100 (73.5)	36 (26.5)	74 (84.0)	14 (16.0)	28 (93.3)	2 (6.7)	30 (78.9)	8 (21.1)	34 (77.2)	10 (22.8)	7 (70.0)	3 (30.0)
Imipenem	124 (91.2)	12 (8.8)	82 (93.2)	6 (6.8)	100.00	0 (0.0)	35 (92.1)	3 (7.9)	34 (77.2)	10 (22.8)	7 (70.0)	3 (30.0)
Polymyxin-B	136 (100.0)	0 (0.0)	88 (100.0)	0 (0.0)	100.00	0 (0.0)	—	—	44 (100.0)	0 (0.0)	10 (100.0)	0 (0.0)
Colistin	136 (100.0)	0 (0.0)	88 (100.0)	0 (0.0)	100.00	0 (0.0)	—	—	44 (100.0)	0 (0.0)	10 (100.0)	0 (0.0)

## Abbreviations

ASM: American Society for Microbiology
ATCC: American Type Culture Collection
CLSI: Clinical and Laboratory Standards Institute
iMLS_B_: Inducible macrolide-lincosamide streptogramin-B
MRSA: Methicillin resistant* Staphylococcus aureus*
MDR: Multidrug resistant.
